# Role of CpxR in Biofilm Development: Expression of Key Fimbrial, O-Antigen and Virulence Operons of *Salmonella* Enteritidis

**DOI:** 10.3390/ijms20205146

**Published:** 2019-10-17

**Authors:** Deeksha Shetty, Juan E. Abrahante, Samuel M. Chekabab, Xuxiaochen Wu, Darren R. Korber, Sinisa Vidovic

**Affiliations:** 1 Department of Food and Bioproduct Sciences, University of Saskatchewan, Saskatoon, SK S7N 5A8, Canada; dms080@mail.usask.ca (D.S.); xuw142@mail.usask.ca (X.W.); drk137@mail.usask.ca (D.R.K.); 2 University of Minnesota Informatics Institute, University of Minnesota, Minneapolis, MN 55455, USA; abrah023@umn.edu; 3 Department of Veterinary and Biomedical Sciences, University of Minnesota, Saint Paul, MN 55108, USA

**Keywords:** *Salmonella* Enteritidis, biofilm development, extracytoplasmic stress response, CpxR, *Salmonella* pathogenicity island

## Abstract

*Salmonella* Enteritidis is a non-typhoidal serovar of great public health significance worldwide. The RpoE sigma factor and CpxRA two-component system are the major regulators of the extracytoplasmic stress response. In this study, we found that the CpxR has highly significant, but opposite effects on the auto-aggregation and swarming motility of *S*. Enteritidis. Auto-aggregation was negatively affected in the ∆*cpxR* mutant, whereas the same mutant significantly out-performed its wild-type counterpart with respect to swarming motility, indicating that the CpxR plays a role in biofilm-associated phenotypes. Indeed, biofilm-related assays showed that the CpxR is of critical importance in biofilm development under both static (microtiter plate) and dynamic (flow cell) media flow conditions. In contrast, the RpoE sigma factor showed no significant role in biofilm development under dynamic conditions. Transcriptomic analysis revealed that the *cpxR* mutation negatively affected the constitutive expression of the operons critical for biosynthesis of O-antigen and adherence, but positively affected the expression of virulence genes critical for *Salmonella*-mediated endocytosis. Conversely, CpxR induced the expression of curli *csgAB* and fimbrial *stdAC* operons only during biofilm development and flagellar *motAB* and *fliL* operons exclusively during the planktonic phase, indicating a responsive biofilm-associated loop of the CpxR regulator.

## 1. Introduction

Non-typhoidal *Salmonella* (NTS) are the leading cause of food-borne gastroenteritis worldwide [1]. Among NTS, *Salmonella enterica* subspecies *enterica* serovar Enteritidis (hereafter referred to as *S*. Enteritidis) is one of the most common serovars associated with human salmonellosis [1]. This zoonotic pathogen owes its great public health importance to an unusually broad host range [2]. According to the World Health Organization, *Salmonella enterica* serotypes Enteritidis and Typhimurium are the two most important serovars of *Salmonella* transmitted from animals to humans [3]. In addition to a large natural reservoir, *S*. Enteritidis has the ability to form robust biofilms on various surfaces, including hydrophobic (e.g., wood and plastic) and hydrophilic surfaces (e.g., glass) under different conditions (e.g., at room temperature under dynamic culture conditions, or at 35 °C under static culture conditions) [4,5], further enhancing its persistence in the environment.

To respond to a wide range of external conditions bacteria must quickly process an external stimulus and adjust their physiology/life style to new conditions or environmental niches. The RpoE sigma factor (σ^E^) and two-component signal transduction system, CpxRA, play crucial roles in maintaining homeostasis of the bacterial envelope [6]. The RpoE regulon consists of a conserved group of functionally coherent genes involved in the synthesis, assembly, and homeostasis of lipopolysaccharides and outer membrane porins, along with a variable group of genes associated with pathogenesis [7]. On the other hand, the Cpx regulon is involved in protein translocation across the inner membrane [8] along with the biogenesis of bacterial appendages [9]. Consequently, these two extracytoplasmic stress-response regulators are involved in the most important biological processes where bacterial cells interact with their surroundings; survival [10,11] and pathogenicity [12,13]. The presence of a wide range of unfolded outer membrane proteins (i.e., any denatured protein) in the periplasmic space of Gram-negative bacteria leads to a cascade of proteolytic steps that results in degradation of the membrane-bound anti-sigma factor, RseA, and release of σ^E^ into the cytosol [14]. Once released into the cytosol, σ^E^-directs RNA polymerase to σ^E^-dependent promoters, thereby inducing the expression of a set of genes (i.e., the RpoE regulon) to protect the cell from the extracytoplasmic stress [7]. The CpxRA extracytoplasmic stress response system includes an inner membrane sensory histidine kinase, CpxA, and a DNA-binding response regulator, CpxR [15]. The presence of misfolded inner membrane proteins leads to autophosphorylation of the cytoplasmic domain of CpxA, which induces a phosphotransfer to the receiver domain of CpxR [16]. Once phosphorylated, CpxR binds cognate DNA, which further results in an expression of the Cpx regulon [16].

It has been shown that the RpoE and Cpx regulators share multiple linkages for inter-extracytoplasmic stress communication in the prokaryotic stress response [6]. Several findings indicate that the Cpx response acts antagonistically to RpoE; specifically, repressing the production of outer membrane β-barrel proteins [17,18] and the σ^E^-induced chaperone Skp [19]. It has been established that the CpxR extracytoplasmic stress response regulator is important in the development of *Actinobacillus pleuropneumoniae* biofilms [20]. In this study, we examined individual and combined contributions of Cpx and RpoE to biofilm-associated phenotypes (i.e., auto-aggregation, swarming motility) as well as biofilm development in *S*. Enteritidis. In addition, we analyzed the transcriptomic responses of *cpxR* mutant and wild-type *S*. Enteritidis cells grown under planktonic and biofilm conditions, enhancing our understanding of the regulatory role of the Cpx regulon during biofilm development.

## 2. Results

### 2.1. Extracytoplasmic Stress-Response Regulators, RpoE and CpxR, Differentially Affect the Growth Rate of S. Enteritidis

**Figure 1:**
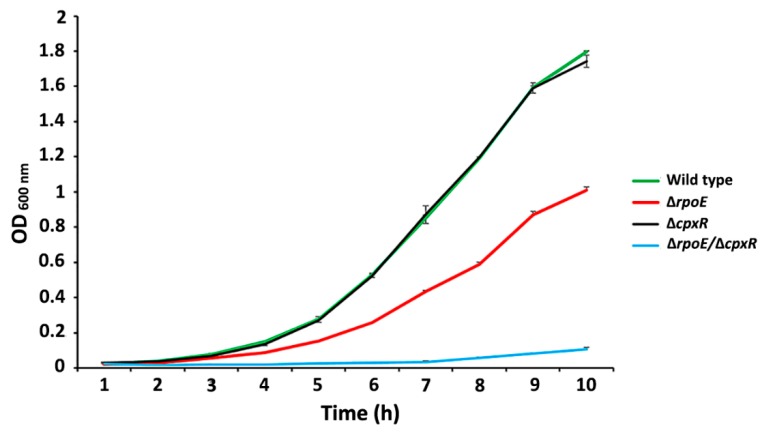
Effect of deletion of the *rpoE, cpxR* and *rpoE/cpxR* genes on the growth of *S*. Enteritidis in Luria–Bertani (LB) broth at 22 °C (room temperature). A significant decrease in growth was seen in Δ*rpoE* as well as for the Δ*rpoE*/Δ*cpxR* strain when compared with the wild type and Δ*cpxR* strains (*p* < 0.001). Error bars represent the standard deviation as determined by *t*-test in comparison with the wild-type and Δ*cpxR* strains.

To investigate the role of the extracytoplasmic regulators, RpoE sigma factor and CpxR system, on the growth rate of *S*. Enteritidis, we measured the growth kinetics of the isogenic Δ*cpxR* and Δ*rpoE* mutants as well as a double Δ*cpxR*/Δ*rpoE* mutant. The growth assay revealed no significant differences in growth kinetics between the wild type and Δ*cpxR* mutant strains (Figure 1).

However, the Δ*rpoE* strain showed an extended lag phase with a final optical density (OD) value approximately 20% (*p* < 0.2) that of the wild-type and Δ*cpxR* mutant strains (specific growth rate ranging from 0.6 [wild type] to 0.48 [Δ*rpoE*] h^−1^; Figure 1). A large, statistically-significant (*p* < 0.05) growth deficiency was observed in the case of the Δ*rpoE*/Δ*cpxR* mutant strain. Deletion of both genes, *rpoE* and *cpxR*, had a much stronger impact on the fitness of *S*. Enteritidis compared to that of the individual deletion mutations (Figure 1).

### 2.2. CpxR Impacts Motility and Auto-Aggregation of S. Enteritidis

**Figure 2:**
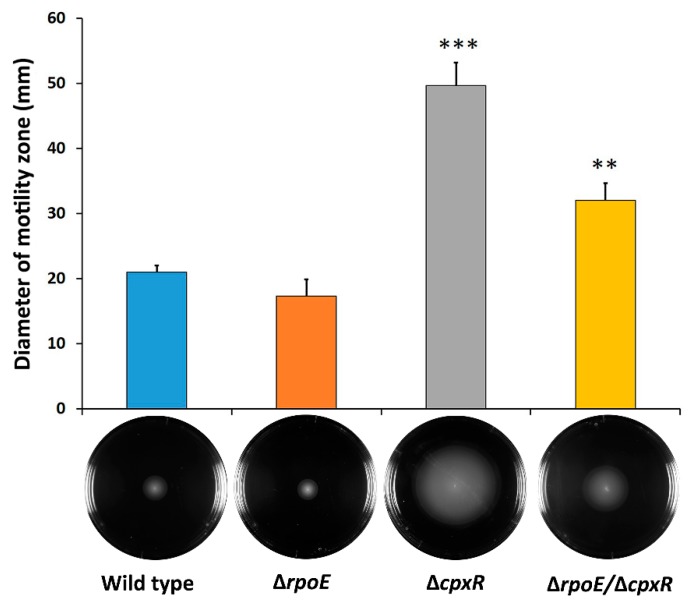
Effect of deletion of the *rpoE, cpxR* and *rpoE/cpxR* genes on the swarming motility, as determined by swimming distance (diameter in mm) of cells in motility agar. Quantification of swarm ring diameter of the wild-type and the isogenic Δ*rpoE,* Δ*cpxR* and Δ*rpoE/cpxR* mutant strains was performed after 24 h of incubation at room temperature. Each data point represents the average of at least three independent experiments. Error bars represent the standard deviation as determined by t-test in comparison to the wild-type strain. ** *p* < 0.01, *** *p* < 0.001.

As the RpoE and CpxR regulate the biogenesis of various outer membrane proteins, as well as the outer membrane itself, we hypothesized that these two regulators would play a role in swarming motility and auto-aggregation, important phenotypic characteristics related to the sessile lifestyle of various bacterial species. Swarming motility of the Δ*rpoE* mutant (17.5 mm in diameter) was slightly-reduced compared to that of the wild-type strain (21 mm in diameter) (Figure 2).

In contrast to the Δ*rpoE* strain, the Δ*cpxR* mutant exhibited more than two times increased swarming (50 mm in diameter) compared to the wild type, while the double Δ*rpoE*/Δ*cpxR* mutant showed a reduced motility trend (32 mm in diameter) compared to the Δ*cpxR,* but increased motility relative to the wild-type strain (Figure 2). Deletion of the *cpxR* gene resulted in a highly-significant (*p* < 0.001) increase in swarming motility of *S*. Enteritidis; whereas, the *rpoE* deletion did not significantly (*p* = 0.3) affect motility of the same organism (Figure 2).

**Figure 3:**
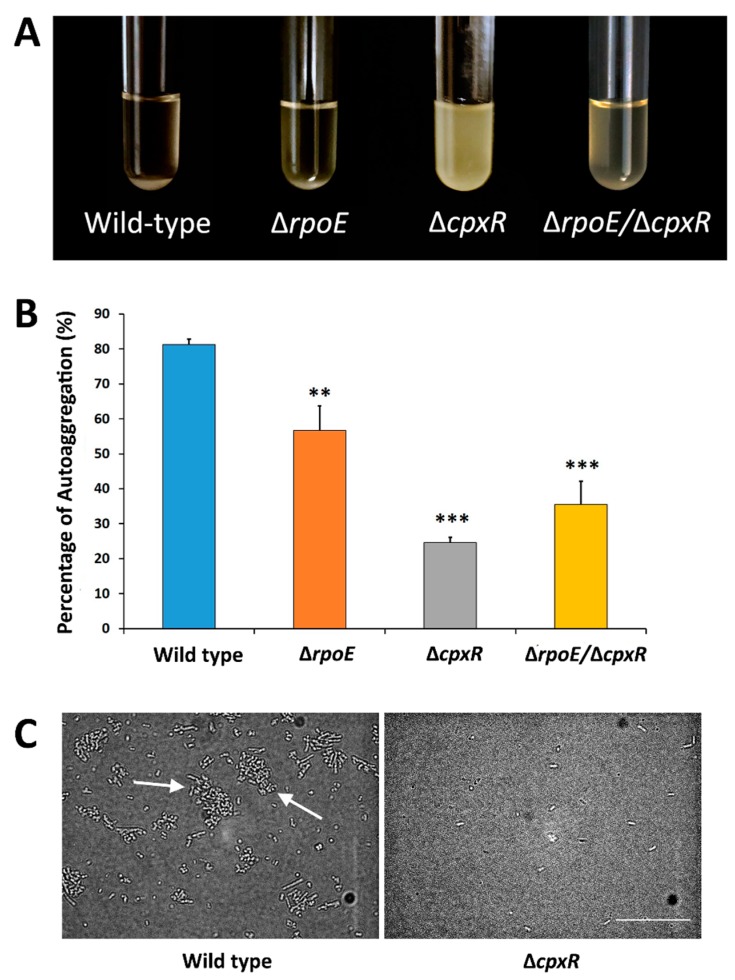
Effect of deletion of the *rpoE, cpxR* and *rpoE/cpxR* genes on the auto-aggregation. **(A**) The Δ*cpxR* cells remained in suspension, while the wild-type cells auto-aggregated and settled to the bottom of the tubes. The other two mutants, the Δ*rpoE* and Δ*rpoE*/*cpxR,* showed intermediate auto-aggregation phenotypes compared to the wild-type and Δ*cpxR* mutant. (**B**) Quantitative measurements of sedimentation-based auto-aggregation assay of the wild-type and mutant strains. Each data point represents the average of at least three independent experiments. The error bars represent the standard deviation determined by t-test on comparison with wild-type. ** *p* < 0.01, *** *p* < 0.001. (**C**) Digital microscope photographs of the wild-type and Δ*cpxR* cells from the auto-aggregation assay, as obtained using phase contrast microscopy and a digital camera. Arrows depicts cell aggregates. Scale bar indicates 20 µm.

We subsequently sought to determine the effect of single gene deletions (*rpoE* and *cpxR*), as well as the effect of the double *rpoE*/*cpxR* deletion, on the ability of *S*. Enteritidis cells to auto-aggregate. Among these four organisms, the wild-type exhibited the greatest ability to auto-aggregate (80%), followed by the Δ*rpoE* mutant (60%) (Figure 3A,B).

In contrast to the wild type, Δ*cpxR* mutant cells showed the weakest auto-aggregation tendencies (22%), virtually resulting in an inability of cells to form aggregates (which subsequently would sediment out) with cells consequently remaining mainly in suspension (Figure 3A,B). Interestingly, the double *rpoE*/*cpxR* deletion mutant resulted in an intermediate phenotype (i.e., a phenotype between the *rpoE* and *cpxR* phenotypes) (Figure 3A). The *cpxR* gene deletion caused a highly-significant (*p* < 0.001) reduction in auto-aggregation, while the *rpoE* gene deletion showed a significant (*p* < 0.01) but less-profound effect compared to deletion of the *cpxR* gene (Figure 3B). In agreement with the auto-aggregation results, direct observation using phase contrast microscopy confirmed the ability of the wild-type strain to form aggregates; whereas, the Δ*cpxR* mutant cells remained mainly in a state of planktonic suspension (Figure 3C). Together, these are compelling data providing initial insights into individual and combined effects of the RpoE and CpxR extracytoplasmic stress response regulators relevant to the sessile lifestyle in *S*. Enteritidis.

### 2.3. CpxR and CpxR/RpoE Have Profound Effects on Biofilm Formation

**Figure 4:**
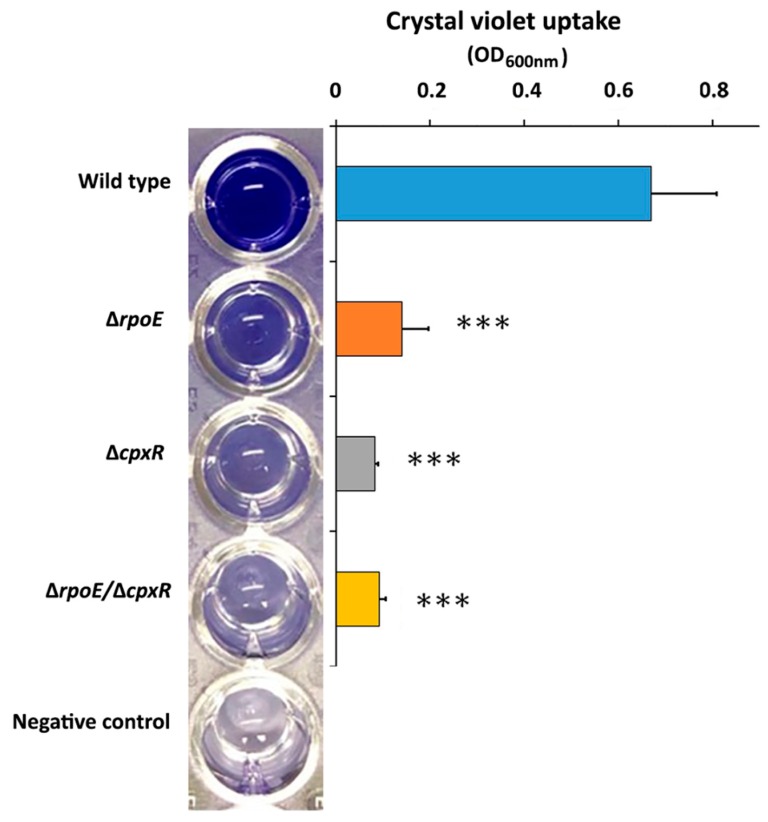
Effect of deletion of the *rpoE, cpxR* and *rpoE/cpxR* genes on the biofilm of *S*. Enteritidis formation under static media condition. Quantification of biofilm production by *S*. Enteritidis wild-type and mutant strains after 48 h growth in tryptic soy broth (TSB) media at 37 °C was determined by measuring adsorption of crystal violet at optical density (OD_600_). *** *p* < 0.001.

Based on the swarming motility and auto-aggregation data, we hypothesized that the *cpxR* and double *cpxR*/*rpoE* deletions would affect biofilm formation in *S*. Enteritidis. To test this hypothesis, we first carried out a microtiter plate biofilm assay, determining the abilities of all three mutants and wild type to form biofilm over a 48 h period. The Δ*cpxR* mutant showed the most significant (*p* < 0.001) reduction in crystal violet uptake (87.7%) compared to the wild type (Figure 4). The double Δ*cpxR/*Δ*rpoE* mutant responded similarly to the Δ*cpxR* mutant, exhibiting a highly-significant (*p* < 0.001) decrease in crystal violet uptake (86.2%), whereas the Δ*rpoE* mutant was slightly less-affected, with a 78.5% decrease in crystal violet uptake compared to the wild type (Figure 4).

**Figure 5:**
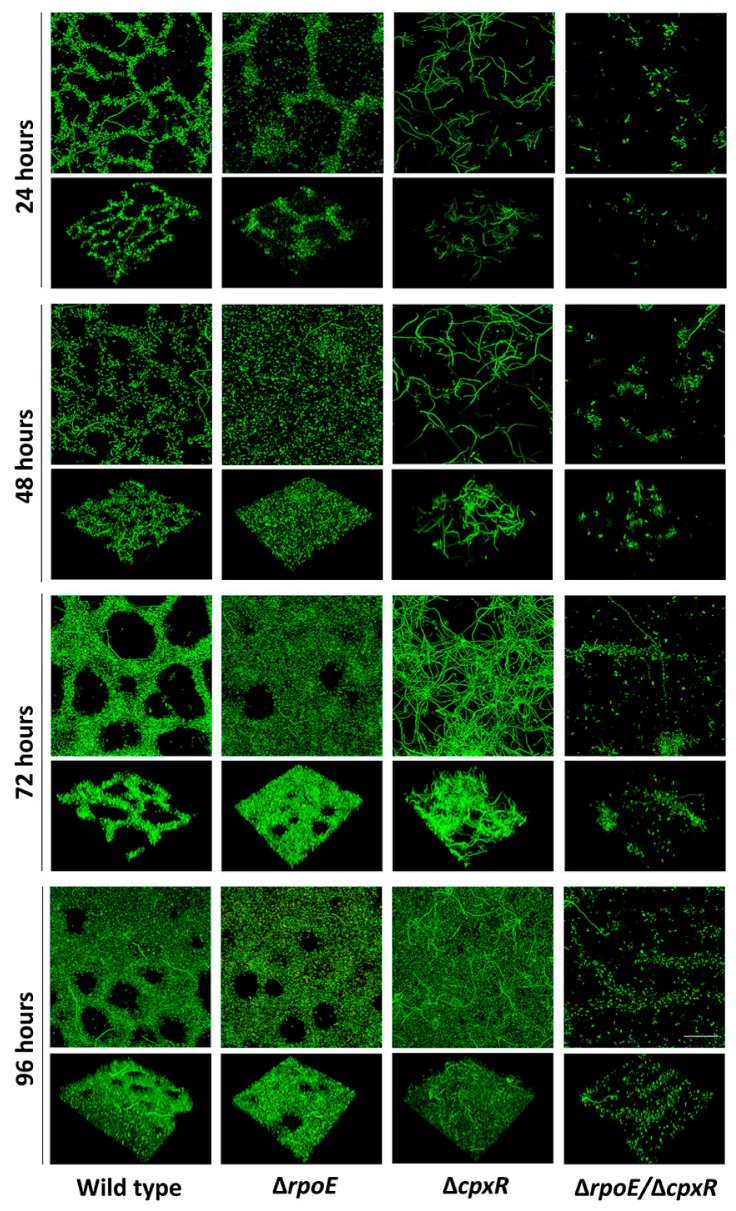
Effect of deletion of the *rpoE, cpxR* and *rpoE/cpxR* genes on the formation of *S*. Enteritidis biofilms under dynamic (flowing) media conditions. Biofilms were grown for 96 h under a continuous nutrient (10% TSB) laminar flow velocity of 0.2 cm sec^−1^ at room temperature. Wild-type and mutant strain biofilms were stained with SYTO 9 and a series of xy CLSM optical thin section (OTS) were obtained at a 0.9 μm interval along the Z-axis. Three-dimensional presentations highlighting differences in biofilm architecture were constructed using a NIS Elements Confocal Microscope Imaging Software (version 4.10). Scale bar indicates 20 µm.

To observe biofilm formation in situ over a longer period of time, and under continuous flow, fully hydrated conditions more representative to real-world settings, we carried out a series of experiments where biofilms were grown in flow cells and then analyzed using non-destructive, confocal laser scanning microscopy (CLSM) over a 96-h time course. After 24 h of incubation, cells of wild-type and the Δ*rpoE* mutant strains were able to attach and develop nascent biofilms, qualitatively characterized as a network of tightly packed microcolonies (Figure 5).

**Figure 6:**
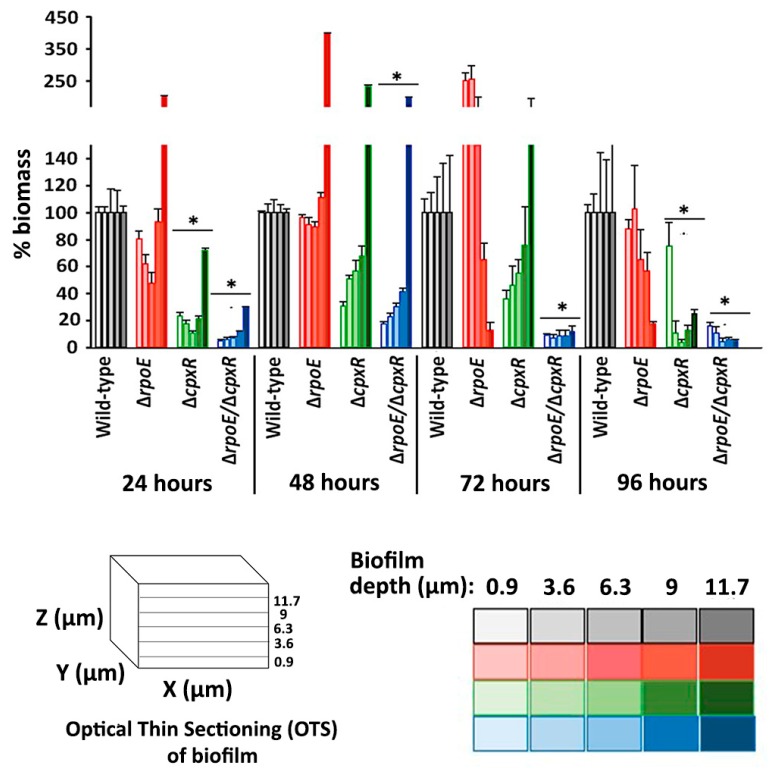
Effect of deletion of the *rpoE, cpxR* and *rpoE/cpxR* genes on the biofilm biomass, as measured by optical thin sectioning (OTS). Biomass of the wild-type, Δ*rpoE*, Δ*cpxR* and Δ*rpoE*/Δ*cpxR* biofilms was measured at 24 h intervals over the duration of the experiment (e.g., 96 h) under conditions reported above (Figure 5 legend). Each measurement was carried out at five OTS depths, including 0.9, 3.6, 6.3, 9 and 11.7 µm, where 0.9 µm sectioning depth represents the biofilm-substratum interface and the 11.7 depth µm represents that region closer to the biofilm-liquid interface (nearest to the center of the flow cell channel). The color-coded table indicates the percentage total biomass relative to the wild-type control biofilm at 0.9, 3.6, 6.3, 9 and 11.7 µm OTS depths, respectively. * *p* < 0.05.

In contrast, the Δ*cpxR* mutant followed a distinct development pathway, forming biofilm consisting of individual, unusually-elongated cells (filaments) that were loosely-attached to the surface, but which didn’t form significant cohesive interactions or inter-cell packing tendencies (Figure 5). The double Δ*rpoE/*Δ*cpxR* mutant resembled the phenotype of biofilms formed by the Δ*cpxR* mutant strain, except that the cells were not elongated (Figure 5). Quantitative biofilm measurements showed that the mean thickness of the Δ*rpoE/*Δ*cpxR* mutant (8 μm) was significantly (*p* < 0.05) reduced from that of the Δ*rpoE* (19 μm), wild-type (18 μm) and the Δ*cpxR* (16 μm) strains (Figure S1). However, the biofilm biomass was significantly-reduced for both the Δ*rpoE/*Δ*cpxR* (*p* < 0.001) and Δ*cpxR* (*p* < 0.005) mutant strains compared to the wild-type (Figure 6).

After 48 h of incubation, the wild type and the Δ*rpoE* mutant formed biofilms where cell aggregates, or microcolonies, merged to become confluent; whereas, the Δ*cpxR* and Δ*rpoE/*Δ*cpxR* mutants retained their initial phenotypes (Figure 5). During this period of time, the thickness of the Δ*cpxR* mutant (35 μm) biofilm significantly (*p* < 0.005) increased compared to the previous day (16 μm); whereas, the thicknesses of biofilms formed by the other three strains only increased slightly (Supplementary Figure S1). However, the biofilm biomass of the Δ*cpxR* mutant was significantly-reduced (*p* < 0.005) compared to that of the wild-type and the Δ*rpoE* mutant strains (Figure 6), indicating an inability of the Δ*cpxR* mutant to form a “mature” fully-developed biofilm, as seen in the wild-type strain after 48 h of incubation. By 72 h, all four organisms maintained their previous biofilm phenotypes (Figure 5). After 96 h of incubation, the Δ*cpxR* mutant exhibited an additional loss of biofilm biomass resulting in a highly significant (*p* < 0.001) decrease in this parameter compared to the wild type (Figure 6), clearly showing a deleterious effect of the single *cpxR* deletion and double *cpxR* and *rpoE* deletions on the ability of *S*. Enteritidis to form extensive, well-developed biofilms as seen in the wild-type organism. The double Δ*rpoE/*Δ*cpxR* mutant showed significantly-less (*p* < 0.001) biofilm biomass compared to that of the wild type during each of the four days of the experiment (Figure 6).

### 2.4. Effect of cpxR Deletion on the Biofilm Transcriptome

During the biofilm formation assays, it was shown that the *cpxR* gene alone, or in a combination with the *rpoE* gene, plays a significant role in biofilm development of *S*. Enteritidis; whereas, the *rpoE* gene alone did not affect biofilm formation of this zoonotic pathogen under dynamic media flow conditions. To further investigate the role of the *cpxR* deletion on biofilm formation, we examined the transcriptional changes that occurred between biofilms of the isogenic Δ*cpxR* mutant and parental wild-type strains. Comparative analysis of the RNA-Seq data showed that 673 genes were significantly differentially expressed by at least 2-fold in all three biological replications of the Δ*cpxR* mutant compared to that of the wild-type. Out of 673 differently-expressed genes in the Δ*cpxR* genetic background, 461 genes were down-regulated (Table S1) and 212 genes were up-regulated (Table S2).

**Figure 7:**
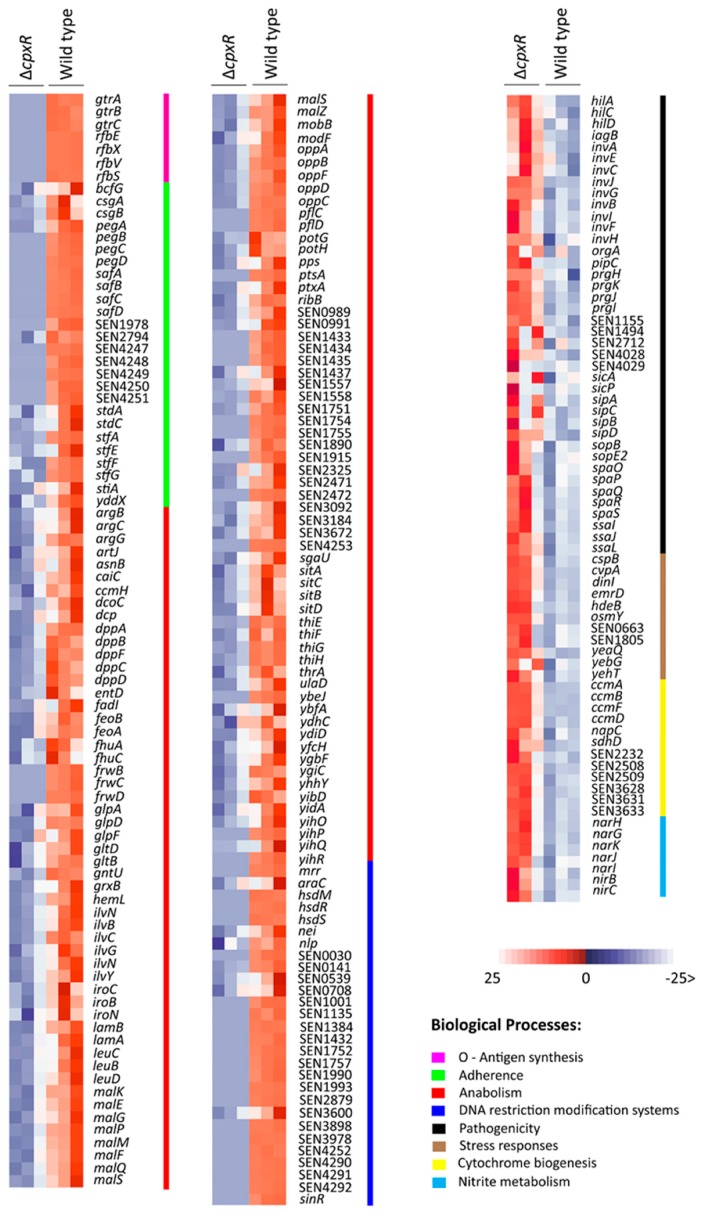
Transcriptional profile of the Δ*cpxR* biofilm relative to its wild-type parental counterpart. RNA was isolated from the 48 h wild-type and Δ*cpxR* mutant strains biofilms. The transcriptomic profiles were determined using a HiSeq 4000 PE100 Illumina platform. A heat map depicting the most impacted (i.e., based on number of genes, level of expression and functional significance to sessile lifestyle) functionally-associated groups of significantly differentially expressed genes (SDEGs) is provided. Each SDEG had to satisfy two criteria: (1) a minimum 2- fold change, and (2) a false discovery rate (FDR) of *p* < 0.05. The intensity of the red and blue colors indicates the fold change in gene expression. Alongside the heat maps are included color-coded lines, further indicating affiliation of SDEGs to different functional groups (e.g., biological processes).

The most notable change caused by deletion of *cpxR* was the down-regulation of entire operons or genes encoding for cell adherence, O-antigen biosynthesis, and anabolic-associated processes (Figure S2 and Figure 7).

The Δ*cpxR* mutant strain manifested a significant decrease in the expression of the *safABCD* operon encoding fimbrial pili, *pegABCD* operon encoding fimbria, *csgAB* operon encoding curli fibers, and *stfAGFE*, as well as *stdAC* encoding fimbrial proteins (Figure 7). Another two operons, *gtrABC* and *rfbVXES*, encoding proteins involved in O-antigen biosynthesis, underwent significant down-regulation in biofilms of the Δ*cpxR* strain compared to wild-type strain biofilms (Figure 7). Besides genes associated with cell appendages and O-antigen biosynthesis, the Δ*cpxR* mutant underwent significant down-regulation of genes encoding for proteins involved in carbohydrate uptake (*malEFKPQSMZG*—maltose metabolic processes; *frwBCD*—phosphotransferase system (PTS fructose-specific transporter), peptide uptake (*oppABCDF*—oligopeptide ATP-binding cassette transporters (ABC transporter); *dppABCDF* – dipeptide ABC transporter), thiamine synthesis (*thiEGHF*), glycolysis (*yihPR*) and leucine synthesis (*leuA*) (Figure 7), clearly indicating that the *cpxR* deletion significantly affected anabolic processes during sessile growth of *S*. Enteritidis.

In contrast to the down-regulation of key genes associated with bacterial appendages, O-antigen biosynthesis and anabolic processes, Δ*cpxR* mutant biofilms caused the up-regulation of numerous virulence-associated genes, including those linked with the type III secretion system (T3SS). Comparative transcriptomics analysis revealed that the Δ*cpxR* biofilm-associated cells significantly up-regulated genes encoding the T3SS apparatus, specifically the needle complex (*invG*, *prgKHIJ, orgA*), an ATPase of T3SS (*invC*) and translocon (*invABEFIJH*, *spaPQROS*) (Figure 7) (Supplementary Table S2). In addition to the T3SS apparatus, the Δ*cpxR* mutant up-regulated a wide range of virulence genes, encoding effectors on both the *Salmonella* pathogenicity island I (SPI1) (*sipABCD*, *sicAP*, *sopBEE2*, *hilACD*, *iagB*, SEN4028, SEN4029) and SPI2 (*ssaIJL*, *pipC*) (Figure 7), suggesting an increased virulence potential of the Δ*cpxR* mutant strain associated with the biofilm phenotype. Besides the large up-regulation of virulence-associated genes, the Δ*cpxR* mutant significantly up-regulated genes associated with stress response (*hdeB*, *cspBH*, SEN0663, *osmY*, *emrD*, *dinI*, *yebG*, *yeaQ*), nitrate respiration (*narJKIHG*) and cytochrome biosynthesis (SEN3633, *ccmABDEF*, SEN3628, *napBC*) (Figure 7), demonstrating that Δ*cpxR* biofilm-associated cells modify their metabolism and adapt to stress as a consequence of *cpxR* gene-deletion. The results of RNA-Seq were validated by real-time polymerase chain reaction (PCR), presented in Figure S3.

### 2.5. Effect of cpxR Deletion on the Transcriptome of Planktonic Cells

In total, 805 genes showed significant (*p* < 0.05) differences in expression between the Δ*cpxR* mutant and wild-type *S*. Enteritidis strains during planktonic growth. The full list of down-regulated and up-regulated genes in the *cpxR* genetic background, along with their gene names, descriptions, biological replications gene expression value, mean gene expression fold-change and false discovery rate *p*-value, are presented in Supplementary Tables S3 and S5, respectively.

In general, the transcriptome of the Δ*cpxR* mutant planktonic cells resembled the transcriptome of the Δ*cpxR* mutant biofilm-associated cells. Comparative expression analysis showed that genes associated with adherence (*safABCD*, SEN4247, SEN4249, *pegABCD*, SEN4251, *stfGF*, SEN4248, SEN4250, SEN1978, *bcfG*, *yddX*, SEN2794), O-antigen synthesis (*rfbEVSX*, *gtrABC*) and anabolic processes (*malEFGKMPQSTZ*, *lamB*, *frwBCD*, *oppABCDF*, *dppABCDF*, *thiEGHF*, *gltBDP*) were significantly down-regulated in planktonic cells of the Δ*cpxR* mutant compared to the wild type (Table S3), further suggesting that down-regulation of these genes was constitutively-regulated due to *cpxR* deletion, but not exclusively linked to the planktonic/sessile mode of life. However, two fimbrial/curli operons, *stdAC* and *csgAB*, exhibited no difference in gene expression between the Δ*cpxR* mutant and wild type during planktonic growth; whereas, these two operons showed significant down-regulation in biofilm-associated Δ*cpxR* mutant cells relative to the wild type. This finding clearly indicates that the *stdAC* and *csgAB* operons are not constitutively-governed by the CpxR regulator, but rather induced in response to the sessile life style of *S*. Enteritidis.

Similarly, the set of up-regulated genes in Δ*cpxR* mutant planktonic cells resembled their respective biofilm transcriptome. In the Δ*cpxR* genetic background during planktonic growth, the genes encoding the T3SS apparatus (*invG*, *prgHIJK*, *orgA*), T3SS specific ATPase (*invC*), translocons (*invABCEFIJH*, *spaOPQRS*), SPI1 effectors (*sipABCD*, *sicAP*, *sopBDEE2*, *hilACD*, *iagB*) and SPI2 effectors (*pipBC*, *ssaBDGHIJKL*, *srfABC*, *sseA*, *avrA*) were significantly up-regulated, showing their constitutive regulation by CpxR. Unique features of the Δ*cpxR* planktonic transcriptome included the significant up-regulation of genes involved in flagellar biosynthesis (*flgABCDEFGHIJKLMN*, *motAB*, *fliAEFGHIJKLMNOPSZ*, *flhBE*) (Table S4). The highly-significant increase (*p* < 0.005) in swarming motility of planktonic Δ*cpxR* cells compared to planktonic wild-type cells could be explained by this unique transcriptional feature of the *cpxR* mutant cells. The results of RNA-Seq were validated by real-time PCR and are presented in Figure S3.

## 3. Discussion

Aggregation, adherence and formation of biofilms in vitro represent important features in the environmental persistence of *S*. Enteritidis [21,22], and consequently influence dissemination of this global pathogen [23,24]. In this study, we found that auto-aggregation was most significantly affected by the *cpxR* deletion. This mutation made it virtually impossible for Δ*cpxR* mutant cells to auto-aggregate and sediment, resulting in the cells remaining suspended in broth media during the auto-aggregation assay. Surprisingly, the same *cpxR* deletion mutation caused the opposite effect with respect to the swarming motility of *S*. Enteritidis. In this case, the isogenic Δ*cpxR* mutant was able to outperform its wild-type parent by more than two times. Thus, our results reveal that the *cpxR* deletion has the highly significant, but opposite effect, on auto-aggregation (i.e., negative) and swarming motility (i.e., positive) in *S*. Enteritidis. Interestingly, the Δ*rpoE* mutant possessed both auto-aggregation and swarming motility phenotypes similar to its wild-type parent, whereas the double Δ*cpxR/*Δ*rpoE* mutant yielded an intermediate phenotype (i.e., phenotypes between the Δ*rpoE* and Δ*cpxR* mutant) with respect to these traits.

Previously, it has been observed that biofilm formation by the same bacterial strain can be influenced by growth under static or dynamic media conditions [25]. To determine the contribution of the RpoE and CpxR, the two major extra cytoplasmic stress response regulators [6], on the formation of *S*. Enteritidis biofilms under static and dynamic conditions, separately we analyzed biofilms grown in microtiter plate (i.e., static condition) and flow cells (i.e., dynamic conditions). Under the static media growth condition all three mutants (Δ*cpxR,* Δ*rpoE*, and Δ*cpxR/*Δ*rpoE*) were significantly affected in their ability to form biofilms compared to that of the wild-type strain. Under flowing (dynamic) conditions, the Δ*rpoE* mutant showed no significant difference in biofilm formation tendencies from the wild-type parent; whereas the double mutant Δ*cpxR/*Δ*rpoE* and Δ*cpxR* mutant exhibited significantly reduced abilities to form biofilms. Our flow cell biofilm assay in conjunction with CLSM imaging and analysis showed that the *cpxR* mutant strain attached poorly to the substratum compared to both the wild-type and the Δ*rpoE* mutant strains. The same mutation caused elongation of cells, which subsequently resulted in formation of a thick, but porous biofilm (i.e., biofilm with low biomass). The double mutation, *rpoE*/*cpxR*, reversed the elongated cell phenotype to the normal wild-type phenotype, albeit with a low number of attached cells, one of the hallmarks of Δ*cpxR* biofilms [26]. Taken together, our study provides evidence that the *rpoE* mutation affects the biofilm formation of *S*. Enteritidis only under the static media condition; whereas, the *cpxR* mutation affects it under both, static and dynamic media conditions. These results can be explained by the fact that under the static condition metabolic wastes accumulate and impose an additional extracytoplasmic stress on the Δ*rpoE* mutant [27], with indirect effect on the ability of this strain to form biofilm. In contrast to the RpoE sigma factor, the CpxR regulator showed its importance in biofilm development under both static and dynamic conditions. This finding clearly indicates that the CpxR does not affect biofilm development via bacterial fitness, but rather directly, by interfering with some steps in the process of biofilm development. It is important to mention that in the all examined biofilm-associated assays (i.e., swarming motility, auto-aggregation and biofilm development under static and dynamic media conditions) the same pattern emerged. The *rpoE* mutant phenotypes were most closely related to the wild-type phenotypes, whereas the *cpxR* mutant showed the most distantly related phenotypes compared to its parental wild type strain. It is unlikely that the growth rate indirectly affected the biofilm-associated phenotypes of the *rpoE* and double mutant. First, the inocula for all four organisms across all assays were standardized. Second, during the swarming motility assay, the double *cpxR*/*rpoE* mutant, despite its growth deficiency, significantly outperformed its parental wild type strain, clearly indicating that this phenotype was not affected by its growth rate but rather by the absence of the *rpoE* and *cpxR* genes.

A number of studies have documented the regulatory role of the CpxR in the synthesis of various bacterial appendages, including Pap pili [28], type IV bundle-forming pili [29,30], P pili [16], Longus [31], and flagella [32], all of which may subsequently affect the biofilm formation. Indeed, several studies have shown that CpxR has a direct [20,33] or indirect [34] role in the biofilm development. Although these studies have given us valuable information on the role of the CpxR on production of various appendages and/or provided information of the involvement of certain genes under CpxR control during biofilm formation, they still haven’t demonstrated an overall regulatory function of CpxR (i.e., regulon) associated with biofilm formation. Accordingly, we first identified the CpxR regulon of *S*. Enteritidis by performing a global transcriptomic analysis of the wild-type planktonic cells compared with their isogenic *cpxR* mutant counterparts. Then, we specifically determined the CpxR biofilm-associated regulon using biofilm transcriptomes of the wild type and the *cpxR* mutant grown under dynamic media conditions.

Most notably, evidence generated through comparison of planktonic and biofilm transcriptomes suggested that the *cpxR* deletion constitutively-affected the expression of operons that are critical for biosynthesis of O-antigen, various adhesins, a large pool of anabolic-associated genes, as well as virulence and stress response genes. The CpxR regulatory circuit also showed a clear division, based on biological process ontology, negatively affecting the expression of adherence- (i.e., O-antigen and adhesins), and anabolic-associated genes; whereas, it positively impacted the expression of the core virulence (i.e., genes encoded on SPI 1 and SPI 2) and stress response genes. It is important to emphasize that the CpxR regulator had a highly-significant and extensive effect on adherence- and virulence-associated genes. For instance, a significant down-regulation (i.e., over 100-fold) of the entire *peg, saf,* SEN4247-SEN4251 fimbrial operons, *gtr,* and *rfb,* as well as O-antigen operons was observed for the isogenic Δ*cpxR* mutant. On the other hand, the Δ*cpxR* mutant showed an extensive up-regulation of salmonellae-essential virulence genes, involving not only genes encoding T3SS, but also genes encoding effector proteins, SopB, SopE2, SipA, SipC, all essential for the invasion of epithelial cells [35]. The extensive up-regulation of essential salmonellae virulence genes in the *cpxR* mutant may be related to the virulence function of some of the down-regulated fimbrial operons. It has been reported that the *S*. Enteritidis *pegD* mutant was reduced significantly in invasiveness in chicken Leghom Male Hepatoma (LMH) cells [36], while host recognition in salmonellae was completely lost without a functional *saf* operon [37]. We hypothesize that by significant down-regulation of fimbrial operons in the Δ*cpxR* background, which are not only involved in adherence, but also in host recognition and invasion, the Δ*cpxR* mutant up-regulates virulence genes essential for the host invasion to compensate for its lack of invasiveness and host recognition.

Besides a large pool of constitutively expressed genes, the CpxR regulator showed responsive control over the flagellar and fimbrial operons, including *fli*, *flg*, *mot*, *flh*; curli *csg* and fimbrial *std* operons. A unique transcriptional feature of the planktonic *cpxR* mutant cells was a significant up-regulation of the flagellar operons, involving genes that encode proteins, MotAB and FliL, which play an essential role in *Salmonella* swarming motility over agar surfaces [38]. Biofilm transcriptomics of the *cpxR* mutant cells showed that the flagellar operons were down-regulated compared to their planktonic counterparts, which is in agreement with the ‘swim-or-stick’ theory. According to this theory, motility and biofilm development are mutually-exclusive processes [39], which can explain down-regulation of the flagellar operons in the *cpxR* biofilm-associated cells. A possible explanation for the up-regulation of the flagellar operons in the *cpxR* mutant planktonic cells compared to the wild-type planktonic cells could be associated with the role of flagella in mechanosensing of surface and initial surface adherence [40]. In another words, the *cpxR* deletion could increase the importance of flagella during surface adherence in the ∆*cpxR* mutant due to inactivation of numerous, normally-functional adhesin operons.

Another unique responsive transcriptional feature, the significant down-regulation of the curli *csgAB* and fimbrial *stdAC* operons, was observed in *cpxR* biofilm-associated cells, suggesting that CpxR regulates expression of these two operons specifically during biofilm development. Curli is the major proteinaceous component of the extracellular matrix in *S. enterica* and plays a very important role in biofilm development during the attachment phase [41], while Std fimbriae play a role in the adhesion of *S. enterica* to specific intestinal receptors of the host [42].

In conclusion, our findings offer new insights into the individual and combined contributions of the major extracytoplasmic stress-response regulators, RpoE and CpxR, during auto-aggregation, swarming motility and biofilm development of *S*. Enteritidis. Although both of these regulators are involved in the extracytoplasmic stress response, our biofilm-related experiments showed the limited importance of the RpoE regulator; whereas, the CpxR protein showed a consistent and significant importance in the all biofilm-related assays. Furthermore, our transcriptomic analyses provided a unique understanding into the regulatory role of the Cpx regulator during biofilm development, indicating that this regulator controls the expression of the most critical fimbrial and O-antigen operons. In addition, this study revealed the global genetic basis underlying CpxR biofilm regulation, which may point to a valuable target(s) for the development of measures to control biofilms generated by this zoonotic pathogen.

## 4. Experimental Procedures

### 4.1. Bacterial Strains, Plasmids and Growth Conditions

*Salmonella enterica* subsp. *enterica* serovar Enteritidis strain ATCC 13076 served as the wild-type organism. Plasmid pKD3 was used as template for amplification of the Cm resistance cassette. Plasmids pKD46 and pCP20 were used during the Red Lambda procedure (see below). Growth media was supplemented with ampicillin (100 μg/mL), chloramphenicol (30 μg/mL) or arabinose 10 mM (Sigma Chemical Co., St. Louis, MO, USA) for maintenance of plasmids and selection of bacterial strains, as required. Bacterial strains were routinely grown in Luria–Bertani (LB) broth with shaking (190 ± 5 rpm) at either 37 or 28 °C, as required.

### 4.2. Construction of ΔcpxR, ΔrpoE and ΔcpxR/ΔrpoE Salmonella Enteritidis Mutant Strains

Construction of chromosomal deletions was performed using the Red Lambda recombination system, as previously described [43]. Briefly, a chloramphenicol resistance cassette, *cat*, flanked by Flp recognition sites, was amplified using the pKD3 plasmid as the DNA template. All primers used for the construction of mutants are listed in Table 1.

Amplified *cat* cassettes were used to transform the wild-type strain harbouring the Red recombination plasmid, pKD46. Introduction of desirable mutations was verified by PCR and DNA Sanger sequencing. To excise the *cat* cassette, a temperature-sensitive, Flp recombinase-expressing vector, pCP20, was introduced via electroporation. Subsequently, the pCP20 plasmid was cured by growing the mutants at elevated temperature (42 °C). All mutants were verified using PCR and Sanger sequencing and later used for functional analyses.

### 4.3. Growth Assay

The differences in the growth kinetics between the wild-type, Δ*cpxR*, Δ*rpoE* and Δ*cpxR/*Δ*rpoE* strains were determined by measuring optical density (OD) at 600 nm over a 10 h time course. Overnight cultures were diluted 100-fold into 100 mL of LB followed by incubation at room temperature (22 °C) with continuous shaking at 190 ± 5 rpm. The specific growth rate (*µ*) was calculated based on the increase in OD_600_ over the optical density interval 0.025 to 0.3 for the wild-type and normalized to the growth rate of the corresponding Δ*cpxR*, Δ*rpoE* and Δ*cpxR/*Δ*rpoE* mutant strains. The formula used to calculate the specific growth rate was *µ* = (ln [OD2-OD1])/(T2-T1) [44].

### 4.4. Swarming-Motility Assay

The swarming motility assay was carried out as described by Legendre et al. [45]. Soft agar (0.25% LB agar) was freshly prepared (i.e., one day prior to the experiment) and used to determine motility of the wild-type and mutant strains. Accordingly, standardized cultures of the wild-type and three mutant strains were stabbed into motility soft agar. Motility diameter was measured as distance in millimeters after incubation for 24 h at room temperature (22 °C).

### 4.5. Auto-Aggregation Assay

Auto-aggregation assays were performed as described by Shanks et al. [46], with minor modifications. Briefly, an aliquot (5 mL) of overnight culture was incubated for 24 h at 22 °C after which the upper 1 mL fraction of the above overnight culture was carefully removed to measure its optical density (OD_600_) (recorded as OD_600_ pre-vortex). The remaining culture in the test tube was then mixed by brief vortexing, followed by optical density measurements (recorded as OD_600_ post-vortex). The “percent aggregation” was calculated using the formula: 100 × (OD_600_ post-vortex − OD_600_ pre-vortex)/OD_600_ post-vortex. Meanwhile, light microscopy digital photographs of the wild-type and Δ*cpxR* strains after auto-aggregation were obtained using a Carl Zeiss Axiokop2 phase contrast microscope equipped with a Carl Zeiss AxioCam ICc1 camera (Zeiss, Jena, Germany) using a 100 times 1.4 N.A. oil immersion lens.

### 4.6. Microtiter-Plate Biofilm Formation Assay

The ability of the wild-type, Δ*rpoE,* Δ*cpxR* and Δ*cpxR/*Δ*rpoE* strains to form biofilms were assessed as previously described [47]. Briefly, overnight cultures of tested strains were diluted 1:100 in tryptic soy broth (TSB) medium, and then used to inoculate the wells of 96-well polyvinyl chloride microtiter plates (Costar 2797, Corning, NY, USA), followed by incubation at 37 °C for 48 h. After incubation, the medium was removed and the wells were washed with 250 μL of sterile distilled water. Subsequently, each well was stained with 250 μL of 0.5% (*w*/*v*) crystal violet for 10 min. After incubation at room temperature, the dye was removed, and the wells washed thoroughly with distilled water followed by air drying. Biofilm accumulation was quantified by solubilizing the bound crystal violet with 250 μL of 30% (*v*/*v*) acetic acid solution, and the absorbance was measured at 600 nm using an automated microtiter plate reader (Packard SpectraCount BS10000 absorbance microplate reader, Cole-Parmer Canada Company, Montreal, QC, Canada).

### 4.7. Flow-Cell Biofilm Formation Assay

Multi-channel flow cells were constructed using polycarbonate sheets into which channels were milled, as described previously [48]. The reactor system consisted a reservoir of sterile medium [10% (*w*/*v*) TSB] connected via silicone tubing to a bubble trap, and subsequently to the flow cell followed by the effluent reservoir. The entire reactor system was sterilized by flushing 5.25% (*w*/*v*) sodium hypochlorite solution for a period of 15 min. Sterile medium was pumped through the flow cell channels, in a once-through fashion, using a Watson–Marlow peristaltic pump (Model 202U; Watson–Marlow, Cornwall, UK). Each flow cell channel was inoculated with 0.5 mL of bacterial culture of an OD_600_ of 0.5 (i.e., mid-log growth phase). The inoculum was retained in the flow cell channel for 30 min. at room temperature (22 °C) to facilitate adhesion of bacterial cells to the flow cell channel surfaces. Flow was then resumed, with biofilms thereafter grown under a continuous nutrient laminar flow velocity of 0.2 cm sec^−1^ at room temperature for the duration of the assay. Biofilms were non-destructively analyzed using a Nikon C2 confocal scanning laser microscope (CSLM; Nikon, Mississauga, ON, Canada) at 24, 48, 72 and 96 h.

### 4.8. Confocal Laser Scanning Microscopy (CLSM) and Biofilm Quantification

Biofilm development was quantified using CLSM (Nikon C2, Mississauga, ON, Canada) and SYTO 9 (Molecular Probes, Life Technologies, Burlington, ON). Biofilm images, corresponding to fluorescence emission in the green (excitation/emission 488/522 nm) (SYTO 9) wavelengths, were acquired in the horizontal (*xy*) and vertical (*xz*) planes. Optical thin section (OTS) stacks were collected over the thickness of the biofilm using a 60 times Plan Apo VC (N.A. 1.4, Nikon) objective lens. Biofilm biomass estimation was carried out by performing image analysis on OTSs using a *z*-step increment of 0.9 µm from the attachment surface (i.e., 0.9, 3.6, 6.3, 9 and 11.7 µm) at five randomly chosen locations per biofilm. The analysis of biofilm biomass (whereby pixels were converted to µm^2^ of cell material) in each OTS was carried out using the Nikon NIS-Elements Confocal Microscope Imaging Software (version 4.10). The biomass percentage at each OTS depth was measured relative to the wild-type using the following formula (mutant/wild-type × 100). The reported mean total biomass was the result of three independent experiments. Biofilm thickness was measured in micrometers (μm) using a computer-controlled, motorized z-axis stepper motor [48,49]. Fifteen random fields were assessed for each biofilm with five separate thickness values obtained per field (*n* = 75). These values were averaged to obtain the thickness data for each biofilm.

### 4.9. Preparation of Planktonic and Biofilm Samples for RNA Extraction

Biomass was collected from biofilms formed by the wild-type and Δ*cpxR* strains after 48 h of incubation using the continuous-flow method described above (e.g., flow cell biofilm assay). Planktonic cells were grown in 10% Tryptic soy broth with shaking (190 ± 5 rpm) at 22 °C until the OD reached 0.5 at 600 nm. Approximately 10^8^ colony-forming units of the wild-type and Δ*cpxR* planktonic cells were accordingly obtained and then centrifuged at 1844× *g* for 10 min. All bacterial samples were re-suspended in RNAprotect bacterial agent (Qiagen, Valencia, CA, USA) and stored at −20 °C until RNA extraction. Total RNA was extracted using the RNeasy mini kit (Qiagen, Valencia, CA, USA) following the manufacturer’s instructions. Sample quality was assessed using capillary electrophoresis (i.e., using an Agilent BioAnalyzer 2100, Santa Clara, CA, USA), generating an RNA Integrity Number (RIN). A RIN of 6 or greater was required to pass initial quality control for sequencing.

### 4.10. RNA-Seq Analysis

After quality control, RNA samples were converted to Illumina sequencing libraries using the KAPA rRNA-depleted (bacteria) stranded library preparation kit (KAPA Biosystems, Wilmington, MA, USA) at the Génome Québec Innovation Centre (Montreal, Quebec, Canada). Approximately 500 nanograms of total RNA was rRNA-depleted using sequence-specific Ribozero capture probes. The mRNA was then fragmented and reverse-transcribed into cDNA. The cDNA fragments were blunt-ended and ligated to indexed (barcoded) adaptors and then amplified using 15 cycles of PCR. Truseq libraries were hybridized to a single-read flow cell and individual fragments were clonally-amplified by bridge amplification on the Illumina cBot. Once clustering was complete, the flow cell was loaded onto a HiSeq 4000 PE100 (Illumina, San Diego, CA, USA) and sequenced using Illumina’s SBS chemistry. The raw reads were first quality-checked with FastQC_v 0.11.7 (http://www.bioinformatics.babraham.ac.uk/projects/fastqc) and then filtered using Trimmomatic v 0.33 [50] to remove low-quality sequences in order to eliminate failed reads. Read mapping was performed via Hisat2 (v2.1.0) [51] using *Salmonella* serovar Enteritidis strain P125109 as reference. Gene quantification was carried out using Cuffquant [52] and Subreads feature counts v1.4.6 [53]. Differentially-expressed genes were identified using the edgeR (negative binominal) feature in CLCGWB (Qiagen, Valencia, CA, USA), followed by filtration based on a minimum 2 times fold-change and false discovery rate (FDR) corrected (*p* < 0.05).

### 4.11. Validation of RNA-Seq Data Using Real-Time Polymerase Chain Reaction (PCR)

The RNA-seq data were validated by quantitative real-time PCR (qRT-PCR). Synthesis of cDNA was carried out using SuperScript™ III Reverse Transcriptase Kit (Invitrogen, Life Technologies, Carlsbad, CA, USA). qRT-PCR was performed on a MiniOpticon^TM^ Real-Time PCR Detection System (Bio-Rad Laboratories Inc., Mississauga, CA, USA) with Quantabio Perfecta SYBR Green FastMix (Qiagen, Valencia, USA). Gene *gyrA*, encoding for DNA gyrase, was selected as an internal reference gene. The expression of *gyrA* in the wild-type and isogenic *cpxR* mutant was not affected by biofilm formation or *cpxR* mutation. Primer sequences for the genes selected for qRT-PCR are listed in Table S5. Results were analyzed using the relative quantification (ΔΔ*C*t) method and expressed as fold-change ± standard error of the mean (SEM) [54].

### 4.12. Experimental Replications and Bioinformatics

All experimental data represent the arithmetic mean of at least three independent experiments. Biofilm thicknesses were analyzed using SAS statistical software (version 9.4, SAS Institute Inc., Cary, NC, USA), and the PROC *t*-test was used to test for significant (*p* < 0.05) differences. Gene Ontology (GO) analysis was conducted using the Database for Annotation, Visualization and Integrated Discovery (DAVID) [55]. To illustrate highly-significant differential gene expression, volcano plots were constructed using R (v 3.4.3) and Plot.ly (v 4.8.0) [56].

## Figures and Tables

**Table 1:** Primers used for construction of mutants.

Primer Name	Sequence (5′-3′)
**Primers for *rpoE* and *cpxR* deletion**	
*rpoE* - Forward	ATG AGC GAG CAG TTA ACG GAC CAG GTC CTG GTT GAA CGG TGT AGG CTG GAG CTG CTT CG
*rpoE* - Reverse	TCA ACG CCT GAT AAG CGG TTG AAC TTT ATT ATC AAT AGC CAT ATG AAT ATC CTC CTT AG
*cpxR* - Forward	ATT AGC GAC GCC TGA TGA CGT AAT TTC TGC CTC GGA GGT ACG TAA ACA TGT AGG CTG GAG CTG CTT CG
*cpxR* - Reverse	CCA GCG TCA ACC AGA AGA TGG CGA AGA TGC GCG CGG TTA AAC TTC CTA CAT ATG AAT ATC CTC CTT AG
**Primers for confirmation of *rpoE* and *cpxR gene* deletions**	
*rpoE* mutant F	GAC CTG TCT ACA ACA TGA CAA ACA
rpoE mutant R	CGG ATC AGG TGA TAA CTC TCC CAG
*cpxR* mutant F	CGC TTG CTC CCA AAA TCT TTT CTG
*cpxR* mutant R	GTT GCT CTA TCA TCA ATC CCT GGC

## Supplementary Materials

Supplementary materials can be found at https://www.mdpi.com/1422-0067/20/20/5146/s1.
