# Supplementary material for: Role of CpxR in Biofilm Development: Expression of Key Fimbrial, O-Antigen and Virulence Operons of Salmonella Enteritidis
Source: Int J Mol Sci. 2019 Oct 17;20(20):5146. doi: 10.3390/ijms20205146 (PMC6829429; doi:10.3390/ijms20205146)
Supplement: Supplementary file 1 [file ijms-20-05146-s001.zip › Supplementary Materials/Supplementary Tables/Table 5 Supplementary Info.docx]

**Supplementary Table 5**. Primers used in Real-Time PCR Gene Expression Assay.

| **Gene name** | **Forward primer 5’-3’** | **Reverse primer 5’-3’** |
| --- | --- | --- |
| *sipB* | TCGCAGCGTCATAAACACTC | TGTTTCGCTGTCTCAACTGG |
| *sicA* | GCCAGTCCCATGGTGTAATC | GATTTGGGATGCCGTTAGTG |
| *hilC* | TCCAGCCCCCATACGTAATA | TGGGTTTGATTTCCTTGAGC |
| *spaP* | ATCGCGATAACCATCCAGAC | CGTCAGGAACCTGTTTCGTT |
| *oppC* | AAAAGCTGGAGGTGGAAGGT | CCCCAGTCGGTATCGAAATA |
| *pegA* | ATAGCGGGCAGAAAAGTTCA | CACTAGCCCAGAAGCGATTT |
| *lppB* | TGCAGTAGTTCTGGGTTCCA | GTTGTCCAGACGCTGGTTCT |
| *thiE* | CAACTCCCGCAATATCCAGT | TGGCCAGTCATATTGAACGA |
| *dppA* | GACATCATCCAGCGGTTTTT | TTGACCGTCTGGTCTTCTCC |
| *gyrA* | ACCGGTACGGTAGGCTTCTT | TGAACGGTTCTTCCGGTATC |
