# Supplementary figures and images for: Role of CpxR in Biofilm Development: Expression of Key Fimbrial, O-Antigen and Virulence Operons of Salmonella Enteritidis
Source: Int J Mol Sci. 2019 Oct 17;20(20):5146. doi: 10.3390/ijms20205146 (PMC6829429; doi:10.3390/ijms20205146)

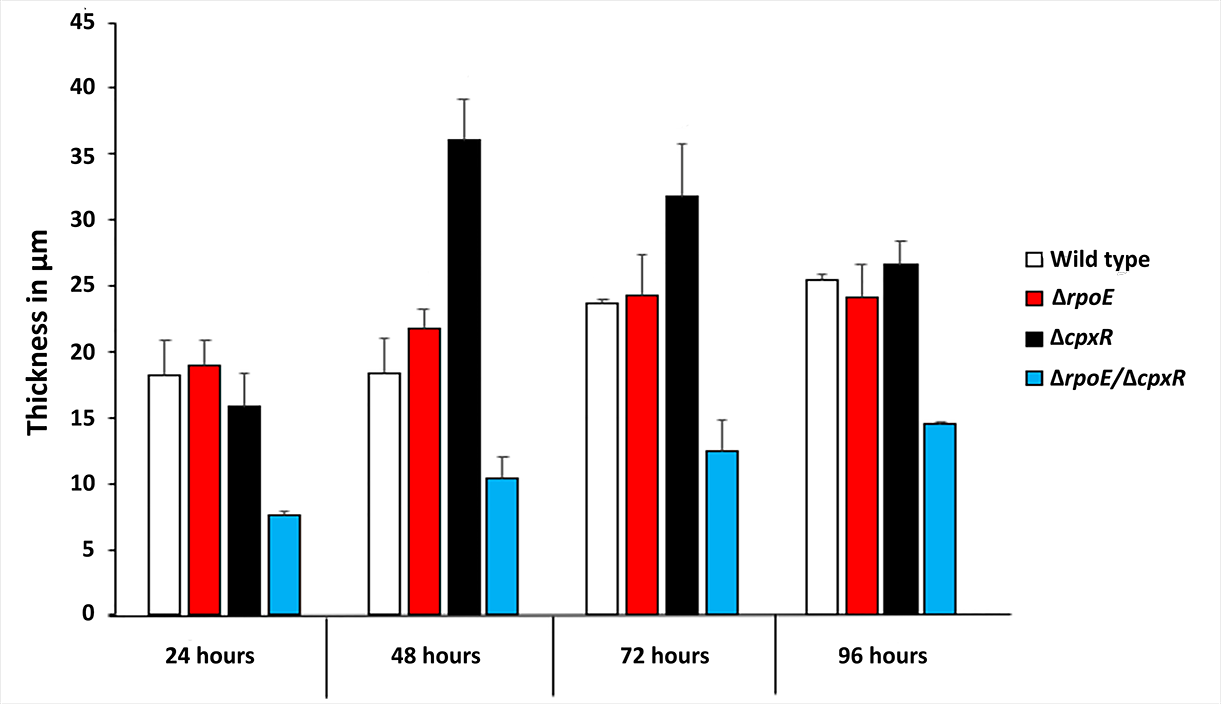

Supplement: Supplementary file 1 [file ijms-20-05146-s001.zip › Supplementary Materials/Supplementary Figures/Supplementary Figure 1.tif]

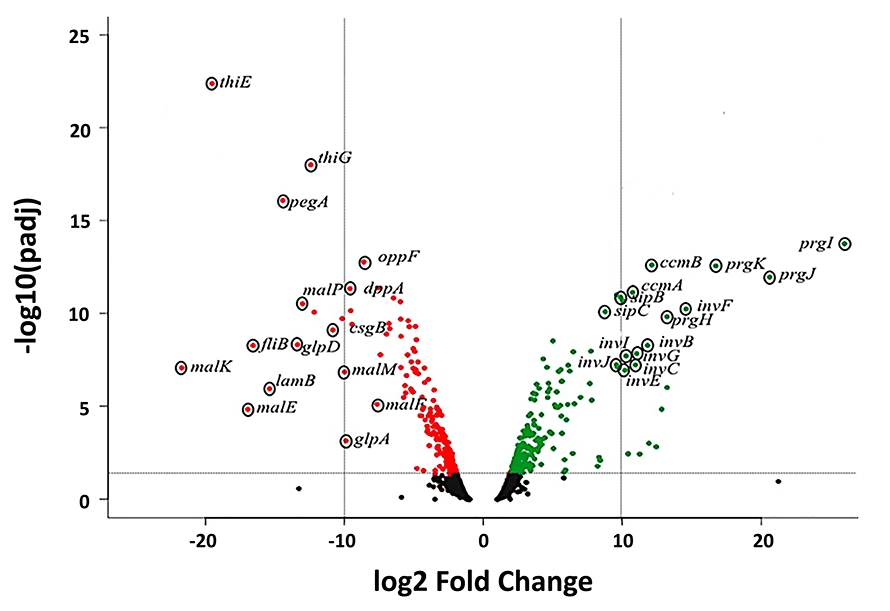

Supplement: Supplementary file 1 [file ijms-20-05146-s001.zip › Supplementary Materials/Supplementary Figures/Supplementary Figure 2.tif]

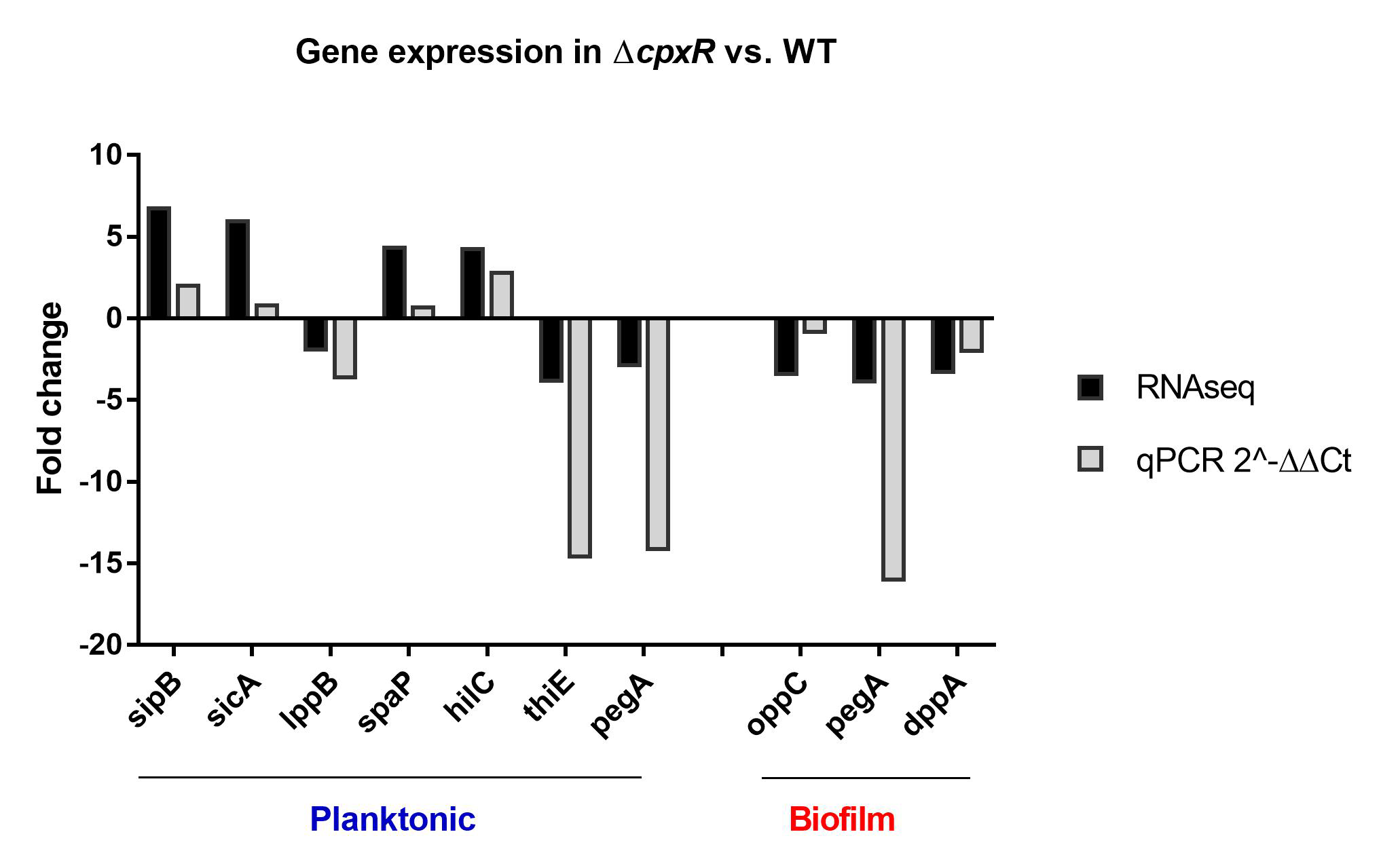

Supplement: Supplementary file 1 [file ijms-20-05146-s001.zip › Supplementary Materials/Supplementary Figures/Supplementary Figure 3.tif]
